# The pons as reference region for intensity normalization in semi-quantitative analysis of brain ^18^FDG PET: application to metabolic changes related to ageing in conventional and digital control databases

**DOI:** 10.1186/s13550-021-00771-0

**Published:** 2021-03-24

**Authors:** A. Verger, M. Doyen, J. Y. Campion, Eric Guedj

**Affiliations:** 1 Department of Nuclear Medicine and Nancyclotep Imaging Platform, Université de Lorraine, 54000 Nancy, France; 2 IADI, INSERM U1254, Université de Lorraine, 54000 Nancy, France; 3 CNRS, Ecole Centrale de Marseille, UMR 7249, Institut Fresnel, Aix-Marseille Université, Marseille, France; 4 CERIMED, Aix-Marseille University, Marseille, France; 5 Department of Nuclear Medicine, Assistance Publique Hôpitaux de Marseille, Timone University Hospital, Marseille, France

**Keywords:** Pons, ^18^FDG PET, Brain, Semi-quantitative analysis, Intensity normalization, Ageing

## Abstract

**Background:**

The objective of the study is to define the most appropriate region for intensity normalization in brain ^18^FDG PET semi-quantitative analysis. The best option could be based on previous absolute quantification studies, which showed that the metabolic changes related to ageing affect the quasi-totality of brain regions in healthy subjects. Consequently, brain metabolic changes related to ageing were evaluated in two populations of healthy controls who underwent conventional (*n* = 56) or digital (*n* = 78) ^18^FDG PET/CT. The median correlation coefficients between age and the metabolism of each 120 atlas brain region were reported for 120 distinct intensity normalizations (according to the 120 regions). SPM linear regression analyses with age were performed on most significant normalizations (FWE, *p* < 0.05).

**Results:**

The cerebellum and pons were the two sole regions showing median coefficients of correlation with age less than − 0.5. With SPM, the intensity normalization by the pons provided at least 1.7- and 2.5-fold more significant cluster volumes than other normalizations for conventional and digital PET, respectively.

**Conclusions:**

The pons is the most appropriate area for brain ^18^FDG PET intensity normalization for examining the metabolic changes through ageing.

## Background

In brain 2-deoxy-2-[^18^F]fluoro-d-glucose (^18^FDG) PET, scaling of tracer uptake to a reference region is essential for data analyses. However, there is currently no genuine recommendation of guidelines for visual or semi-quantitative ^18^FDG PET analyses [1, 2]. Proportional scaling [3], i.e., intensity normalization based on the whole-brain, and that related to specific brain areas, such as the cerebellum [4] and the pons [5], have been proposed, especially for the diagnosis of neurodegenerative disorders. Nevertheless, proportional scaling intensity normalization is biased in cases of diffuse hypometabolism leading to artefactual hypermetabolism [6]. The choice of other specific brain regions assumes that this reference region for intensity normalization is not physio-pathologically affected.

The gold standard method for brain analysis remains the absolute quantification of brain glycolytic metabolism, but it is principally used for research purposes, and its non-applicability in routine is related to the invasive measures of radioactivity determined by sampling arterial blood [7]. The best way to define a reference region for semi-quantitative analysis could be based on these absolute quantification studies, which showed that the metabolic changes related to ageing affect the quasi-totality of brain regions in healthy subjects [7].

Meanwhile, digital PET technology provides significant advances in the quality of brain ^18^FDG PET images mainly through improvements in spatial resolution that could modify the choice of the reference region for intensity normalization with a better visualization of small anatomical structures [8]. For this purpose, we propose to define the most appropriate region for intensity normalization in semi-quantitative analysis of brain ^18^FDG PET given the well-known physiological metabolic changes related to ageing in two different populations of healthy controls who underwent brain ^18^FDG PET with conventional and digital PET systems.

## Methods

### Populations

Two populations of healthy controls who received a brain ^18^FDG PET scan on a conventional system (Discovery ST, GE Healthcare^®^, APHM, La Timone, Marseille, France) or a digital system (Vereos, Philips^®^, CHRU Nancy, France) and who were free from neurological and psychiatric diseases as well as any systemic pathology or drugs interfering with brain metabolism were selected for this study (respective Clinical Trials Ref: NCT00987090 and NCT03345290).

### Brain ^18^FDG PET acquisition and reconstruction

2-[^18^F]FDG was injected intravenously (150 MBq for the conventional system and 2–3 MBq/kg for the digital camera), while the subjects were in a resting state with their eyes closed in a quiet environment as recommended [1]. Image acquisition started 30–45 min after injection and ended 15 min later [1]. All PET images were reconstructed with iterative OSEM methods, as performed in clinical practice, and corrected for scatter, random and attenuation with a CT scan.

### Statistical and SPM analyses

The ^18^FDG PET brain images were pre-processed using SPM12 (Wellcome Department of Cognitive Neurology, Institute of Neurology, London, UK) running on MATLAB 2018a (MathWorks Inc., Sherborn, MA). After an initial step of approximate manual reorientation and positioning to the MNI space, the spatial normalization of each PET image into the MNI space was performed using the MNI template for the conventional camera and an adaptive template for the digital camera. PET images were then smoothed with a post-Gaussian filter adapted to the spatial resolution of the system (8 mm of smoothing for the conventional camera and 4 mm for the digital camera). Marsbar software (http://marsbar.sourceforge.net/) was used to extract the metabolism values of the 116 areas of the Automated Anatomical Labelling (AAL, [9]) atlas in addition to the whole grey matter, midbrain, pons and medulla provided by Pickatlas (https://www.nitrc.org/projects/wfu_pickatlas/) at the individual level.

Pearson coefficient correlations were determined between each of the 120 brain atlas regions and age using the 120 brain atlas regions for intensity normalization. The median and maximal correlation coefficients with age for each intensity normalization region were reported for both the conventional and digital PET systems. A focus was placed on the regions for which the medians of Pearson correlation coefficients with age were less than − 0.5.

Then, these reference regions were used for intensity normalization [3]. PET images were normalized in intensity by dividing PET images with individual values of brain areas aforementioned and derived from the AAL atlas gathering all areas belonging to a defined brain structure when feasible (e.g., the cerebellum lobes and vermis) to be the closest possible to the routine practice. Visual inspections of the images at the different stages of the pre-processing procedure ensured the quality and convergence of the different methods applied. The metabolic changes related to ageing were assessed through negative linear correlations, with age and sex added in the model as covariates for each intensity normalization (*p* < 0.05 FWE corrected for the conventional PET system and equivalent corresponding T-voxel level for the digital PET system, in order to take into account the distinct number of subjects in the two database that are thus analysed using the same T-score threshold).

## Results

### Population

The healthy control population whose scans were acquired on the conventional camera consisted of 56 subjects [50 ± 17 (min: 21; max: 78), years old, 33 women], whereas 78 controls [58 ± 16 (min: 22; max: 87), years old, 42 women] had their scans performed on the digital system.

### Results provided by the different intensity normalization regions

The correlation analyses between each of the 120 brain atlas regions and age using the 120 brain atlas regions for intensity normalization are summarized in Table 1. The vermis 8 and the cerebellum 8L were the brain atlas regions showing the highest median correlation coefficients of − 0.51 and − 0.63 for the conventional and digital PET, respectively. Their respective maximal correlation coefficients were − 0.76 for the vermis 8 region with conventional PET and − 0.82 for the cerebellum 8L region with digital PET. Interestingly, among the 120 atlas regions used for PET intensity normalization, only the pons in addition to the cerebellum showed median correlation coefficients below − 0.5 (maximal correlation coefficient of − 0.82) for digital PET.

**Table 1:** Median and highest Pearson correlation coefficients (provided from correlation coefficients of the 120 brain regions) between PET metabolism and age for each region of intensity normalization in both conventional and digital PET systems

Region for intensity normalization	Conventional PET		Digital PET	
	Strongest correlation coefficient	Median of correlation coefficients	Strongest correlation coefficient	Median of correlation coefficients
Precentral_R	− 0.573688445	0.279184148	− 0.581382052	0.293280215
Precentral_L	− 0.622302284	0.232181965	− 0.628568612	0.266985128
Frontal_Sup_R	− 0.600980191	0.319870899	− 0.690629185	0.242280289
Frontal_Sup_L	− 0.547693637	0.405334595	− 0.599914817	0.443110434
Frontal_Sup_Orb_R	− 0.629579861	0.084998269	− 0.700581666	− 0.108439103
Frontal_Sup_Orb_L	− 0.547437357	0.192820744	− 0.703649178	− 0.033764917
Frontal_Mid_R	− 0.553053145	0.349833531	− 0.584128133	0.463018236
Frontal_Mid_L	− 0.551382331	0.315606715	− 0.610512904	0.39907714
Frontal_Mid_Orb_R	− 0.55913071	0.098969813	− 0.684911592	− 0.036623595
Frontal_Mid_Orb_L	− 0.445475539	0.255947537	− 0.680085738	0.1323272
Frontal_Inf_Oper_R	− 0.403554772	0.451057221	− 0.584413796	0.431311318
Frontal_Inf_Oper_L	− 0.346777766	0.545425764	− 0.542003107	0.481079751
Frontal_Inf_Tri_R	− 0.549174379	0.296954014	− 0.608717652	0.267245207
Frontal_Inf_Tri_L	− 0.558387379	0.286348933	− 0.653848914	0.273189928
Frontal_Inf_Orb_R	− 0.52705298	0.333803877	− 0.6524112	0.21577136
Frontal_Inf_Orb_L	− 0.464517096	0.368956454	− 0.628902165	0.32330541
Rolandic_Oper_R	− 0.566697841	0.243996131	− 0.678571105	0.095847239
Rolandic_Oper_L	− 0.618467447	0.211708259	− 0.723144101	− 0.112062758
Supp_Motor_Area_R	− 0.517843026	0.180772399	− 0.460519688	0.426544183
Supp_Motor_Area_L	− 0.696169862	− 0.02036436	− 0.73268581	0.039897831
Olfactory_R	− 0.388765983	0.487926686	− 0.657210451	0.105375048
Olfactory_L	− 0.528013879	0.384147962	− 0.721491098	− 0.122749257
Frontal_Sup_Medial_R	− 0.358836732	0.524163102	− 0.488283894	0.489288049
Frontal_Sup_Medial_L	− 0.573032908	0.423672249	− 0.735839552	0.25158455
Frontal_Med_Orb_R	− 0.484358087	0.39321911	− 0.615616599	0.337668074
Frontal_Med_Orb_L	− 0.50075176	0.376511335	− 0.74510263	0.041807974
Rectus_R	− 0.55071263	0.351304466	− 0.635567564	0.035246613
Rectus_L	− 0.604668295	0.259112277	− 0.671649932	− 0.154421386
Insula_R	− 0.626506716	0.161993577	− 0.734040284	− 0.072023968
Insula_L	− 0.443959797	0.496975678	− 0.56772327	0.513856619
Cingulum_Ant_R	− 0.275972126	0.634247449	− 0.400842278	0.640119849
Cingulum_Ant_L	− 0.692031912	0.350939125	− 0.78453279	0.189981663
Cingulum_Mid_R	− 0.615009986	0.262411063	− 0.579931165	0.467563092
Cingulum_Mid_L	− 0.664484269	0.167834424	− 0.730987309	0.232708068
Cingulum_Post_R	− 0.526277264	0.019893397	− 0.624772162	0.120155957
Cingulum_Post_L	− 0.555623248	0.026567322	− 0.634707019	0.240298045
Hippocampus_R	− 0.74488582	− 0.158224441	− 0.776691477	− 0.178442999
Hippocampus_L	− 0.792432684	− 0.401600367	− 0.794914907	− 0.355792507
ParaHippocampal_R	− 0.547796599	0.135694163	− 0.741894049	− 0.03170524
ParaHippocampal_L	− 0.691581684	− 0.020746522	− 0.774862984	− 0.184001347
Amygdala_R	− 0.690685381	− 0.050409819	− 0.738301387	− 0.264093722
Amygdala_L	− 0.675326502	− 0.09187106	− 0.738046871	− 0.344906004
Calcarine_R	− 0.597218653	0.004930533	− 0.59736577	0.209424087
Calcarine_L	− 0.641938573	− 0.076213494	− 0.693935653	− 0.086552086
Cuneus_R	− 0.726146822	− 0.154685879	− 0.621177518	0.096712726
Cuneus_L	− 0.785087897	− 0.348003457	− 0.666285592	− 0.124622161
Lingual_R	− 0.634614302	− 0.097770865	− 0.643697071	− 0.046781064
Lingual_L	− 0.685486599	− 0.166388559	− 0.670109744	− 0.04623892
Occipital_Sup_R	− 0.701641346	− 0.194678777	− 0.646231705	− 0.064989794
Occipital_Sup_L	− 0.741727211	− 0.306092732	− 0.597199679	0.127544711
Occipital_Mid_R	− 0.727813649	− 0.364255804	− 0.647178347	− 0.059276144
Occipital_Mid_L	− 0.718730655	− 0.244437115	− 0.672193986	0.035856165
Occipital_Inf_R	− 0.723331225	− 0.386129463	− 0.668353467	− 0.211046407
Occipital_Inf_L	− 0.745424761	− 0.389693675	− 0.689600876	− 0.207004321
Fusiform_R	− 0.726536142	− 0.195906801	− 0.760147469	− 0.312002428
Fusiform_L	− 0.737547599	− 0.265574857	− 0.756777592	− 0.30482379
Postcentral_R	− 0.709883189	0.024251053	− 0.626583194	0.099793863
Postcentral_L	− 0.76459292	− 0.110140116	− 0.734129726	− 0.048775906
Parietal_Sup_R	− 0.710966862	− 0.163768876	− 0.600734839	0.175798905
Parietal_Sup_L	− 0.639227666	− 4.90902E − 05	− 0.542532856	0.337939446
Parietal_Inf_R	− 0.620798761	0.113031765	− 0.541267618	0.442951513
Parietal_Inf_L	− 0.554435839	0.25511845	− 0.519628	0.459809369
SupraMarginal_R	− 0.642901203	0.143077102	− 0.631066657	0.16035289
SupraMarginal_L	− 0.67010586	0.198101079	− 0.629274111	0.305772909
Angular_R	− 0.637290184	− 0.021187495	− 0.576971854	0.223955824
Angular_L	− 0.611346979	0.099585441	− 0.577059191	0.293618718
Precuneus_R	− 0.745445943	− 0.14394911	− 0.687422118	0.094901913
Precuneus_L	− 0.757202509	− 0.219708792	− 0.708572999	0.033021343
Paracentral_Robule_R	− 0.778348539	− 0.344721681	− 0.702759859	− 0.103953847
Paracentral_Robule_L	− 0.784491443	− 0.417984838	− 0.794021505	− 0.424682684
Caudate_R	0.307397852	0.497272916	0.398152235	0.640465612
Caudate_L	− 0.524293718	0.387711671	− 0.711582026	0.385198422
Putamen_R	− 0.602942275	0.125257327	− 0.701652279	− 0.198897079
Putamen_L	− 0.630316648	− 0.007083048	− 0.70161431	− 0.11815898
Pallidum_R	− 0.510633293	0.20883992	− 0.770671936	− 0.407366194
Pallidum_L	− 0.448907585	0.271878766	− 0.709597016	− 0.223370032
Thalamus_R	− 0.507908201	0.263313022	− 0.67710738	0.302515492
Thalamus_L	− 0.654566592	− 0.060126393	− 0.74406809	− 0.109863951
Heschl_R	− 0.485771916	0.298329103	− 0.55866801	0.43147372
Heschl_L	− 0.472564385	0.35518222	− 0.503638686	0.575685721
Temporal_Sup_R	− 0.625069091	0.254407932	− 0.624234384	0.348479101
Temporal_Sup_L	− 0.68587206	0.168621993	− 0.652154125	0.302844751
Temporal_Pole_Sup_R	− 0.331232941	0.425860523	− 0.44600523	0.406669675
Temporal_Pole_Sup_L	− 0.363542986	0.354619085	− 0.500438422	0.380854532
Temporal_Mid_R	− 0.690671811	0.044779431	− 0.658061148	0.11769236
Temporal_Mid_L	− 0.697074384	0.064873746	− 0.638795334	0.231016521
Temporal_Pole_Mid_R	− 0.451855567	0.225791623	− 0.644696334	0.035810993
Temporal_Pole_Mid_L	− 0.457567288	0.245337503	− 0.704001434	0.029743346
Temporal_Inf_R	− 0.68436998	− 0.104670403	− 0.696015575	− 0.063048755
Temporal_Inf_L	− 0.669764664	− 0.031455074	− 0.689992535	0.035977061
Cerebelum_Crus1_R	− 0.600476473	− 0.004144622	− 0.64067283	− 0.112577583
Cerebelum_Crus1_L	− 0.666707111	− 0.13413203	− 0.68045527	− 0.193184819
Cerebelum_Crus2_R	− 0.681004608	− 0.302210346	− 0.679835366	− 0.196911931
Cerebelum_Crus2_L	− 0.687555117	− 0.232091696	− 0.684717528	− 0.150689558
Cerebelum_3_R	− 0.631993976	− 0.168105517	− 0.69448275	− 0.107741771
Cerebelum_3_L	− 0.576269744	− 0.009951379	− 0.647040809	− 0.007816927
Cerebelum_4_5_R	− 0.650573984	− 0.181355372	− 0.687937783	− 0.21895484
Cerebelum_4_5_L	− 0.663071101	− 0.077466212	− 0.730497017	− 0.123468168
Cerebelum_6_R	− 0.677921489	− 0.155131146	− 0.710308279	− 0.303651351
Cerebelum_6_L	− 0.735262898	− 0.279267569	− 0.716515286	− 0.28440532
Cerebelum_7b_R	− 0.704269564	− 0.332758809	− 0.738855392	− 0.400111216
Cerebelum_7b_L	− 0.743454724	− 0.403805093	− 0.729542146	− 0.333328681
**Cerebelum_8_R**	− 0.729481495	− 0.437717362	− 0.812531147	** − 0.61846924**
**Cerebelum_8_L**	− 0.753248363	** − 0.500101319**	− 0.823696914	** − 0.630973003**
**Cerebelum_9_R**	− 0.695106139	− 0.36420094	− 0.79927817	** − 0.503436315**
**Cerebelum_9_L**	− 0.744673144	− 0.476228365	− 0.811756228	** − 0.579151524**
Cerebelum_10_R	− 0.548985851	− 0.118360681	− 0.717244723	− 0.333550516
Cerebelum_10_L	− 0.571047001	− 0.201373641	− 0.541209852	0.039102255
Vermis_1_2	− 0.644498893	− 0.180633313	− 0.732799769	− 0.208601441
Vermis_3	− 0.584677906	− 0.134242733	− 0.671825282	− 0.096599336
Vermis_4_5	− 0.577923911	− 0.137586851	− 0.642737286	− 0.089295963
Vermis_6	− 0.531525033	− 0.04047739	− 0.610495443	− 0.101942777
Vermis_7	− 0.578924525	− 0.093968004	− 0.662218149	− 0.224039981
**Vermis_8**	− 0.756376798	** − 0.506853104**	− 0.790218976	** − 0.616443676**
Vermis_9	− 0.742895596	− 0.469781549	− 0.753866317	− 0.457157983
Vermis_10	− 0.667363951	− 0.342948819	− 0.714105274	− 0.232058498
Midbrain	− 0.71649863	− 0.13811898	− 0.748687248	− 0.13147618
**Pons**	− 0.718410905	− 0.385970718	− 0.818592258	** − 0.547892778**
Medulla	− 0.737949443	− 0.406080404	− 0.776357698	− 0.371013333
Whole grey-matter	− 0.738042818	0.124152213	− 0.734258642	0.16754782686138

Regions with median correlation coefficients less than − 0.5 are highlighted in bold

**Fig. 1:**
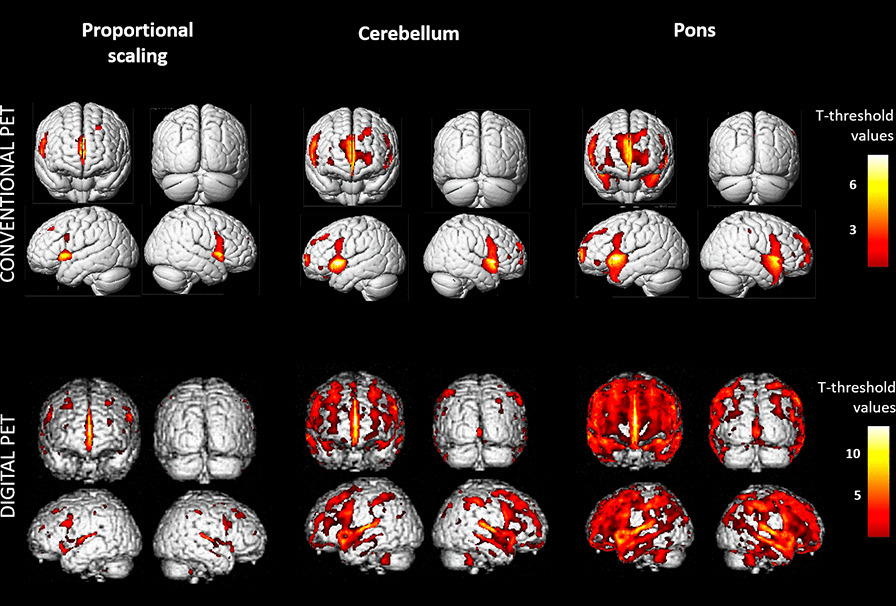
Metabolic changes related to ageing with SPM linear regression analyses among the intensity normalization methods based on the whole grey-matter, the cerebellum and the pons for the conventional camera (FWE, *p* < 0.05, upper panel) and the digital camera (equivalent T-voxel level of the previous conventional analysis, lower panel). The results are represented with 3D-rendered volumes

As depicted in Fig. 1, the intensity normalization by the pons was, however, the best region after SPM linear regression analyses for both cameras with respective significant cluster volumes of 143,330 (T-max voxel at 10.2) and 453,080 (T-max voxel at 13.5) mm^3^ for conventional and digital PET scanners. By comparison, these linear regression analyses revealed only 41,528 (T-max voxel at 8.3) and 84,216 (T-max voxel at 9.8) mm^3^ significant cluster volumes for the whole grey-matter and cerebellum intensity normalization, respectively, for the conventional system and 63,079 (T-max voxel at 12.2) and 183,378 (T-max voxel at 12.6) mm^3^ significant cluster volumes, respectively, for the digital system.

## Discussion

The present study shows that the pons is the best brain region for intensity normalization of brain ^18^FDG PET scans for the detection of metabolic changes related to ageing. These findings are reinforced by the fact that our results are duplicated in two independent populations of healthy controls with both conventional and digital PET systems.

Metabolic changes related to ageing, which are physiological changes that have been widely studied, have been highlighted in well-conducted studies involving absolute glycolytic quantification, which remains the gold standard for analysis of brain ^18^FDG PET images [7]. Even if these age-related changes affect the quasi-totality of brain areas [7], a more pronounced age-related effect is visualized in the frontal and temporal regions (Fig. 1), which is in accordance with previously reportss [10, 11]. Interestingly, our results indicate that the most suitable regions for intensity normalization of brain ^18^FDG PET scans are the pons and the cerebellum (Table 1), these two regions being known to be poorly affected by age-related changes. Among both regions, intensity normalization by the pons exhibits the best performances after SPM analyses (Fig. 1). This region has previously been proposed as a reference for the detection of Alzheimer’s disease [5]. The normalization by the pons region allows to highlight subtle changes of ageing described in studies with absolute quantification and should thus be the more appropriate region for semi-quantification of brain ^18^FDG PET images. As long as the pons is free of any pathological involvement, this region should be recommended for visual as well as semi-quantitative analyses of brain ^18^FDG PET imaging related to other conditions, even though further studies are needed to translate our results to a patient group approach. Conversely, using the pons for intensity normalization of ^18^FDG PET images requires to imperatively correct the statistical models from the age covariable.

Histogram-based methods have been also recently proposed to improve the intensity PET normalization. These results were, however, obtained in healthy subjects with artificial introduced hypometabolisms [12]. These data-driven methods need group of patients, whereas the pons allows the intensity normalization of brain PET images at the individual level, easily applicable for the visual analysis in clinical routine. Targeting a specific brain region for normalization is nevertheless subject to the fact that we must precisely know brain areas affected by the pathology, which could be in some cases a limitation.

Our results are strengthened by the fact that they have been obtained twice, after regional correlation analyses and voxel-to-voxel analyses. Moreover, these results are visualized in two different populations for which brain ^18^FDG PET scans have been acquired with two different PET technology systems.

Of note, more significant results were observed when using the population having performed a brain ^18^FDG PET with the digital camera than those having performed brain PET images with the conventional system (Table 1 and Fig. 1). These results are in accordance with the higher performance parameters provided with the new digital PET systems when compared to conventional PET systems [8], even if the spatial normalization method and post-filter smoothing of images have been adapted for digital PET technology. This highlights that digital PET technology modifies the results of PET intensity normalization with its ability to delineate small anatomical structures such as the pons.

## Conclusions

In conclusion, the current study proposes to use the pons as a reference region for intensity normalization of semi-quantitative analysis of brain ^18^FDG PET with both conventional and digital PET technologies. This method is reported for the detection of metabolic changes related to ageing but should be proposed for visual as well as semi-quantitative analyses of brain ^18^FDG PET imaging related to other pathophysiological mechanisms as long as the pons region is free of any pathological involvement. Further studies are needed to translate these results to a patient group approach.

## Data Availability

The data that support the findings of this study are available from the corresponding author upon reasonable request.
